# Identification of inhibitors against α-Isopropylmalate Synthase of Mycobacterium tuberculosis using docking-MM/PBSA hybrid approach

**DOI:** 10.6026/97320630013144

**Published:** 2017-05-31

**Authors:** Preeti Pandey, Andrew M. Lynn, Pradipta Bandyopadhyay

**Affiliations:** 1 School of Computational and Integrative Sciences, Jawaharlal Nehru University, New Delhi, INDIA 110067

**Keywords:** α-Isopropylmalate Synthase, Mycobacterium tuberculosis, docking-MM/PBSA hybrid

## Abstract

α-Isopropylmalate Synthase (α-IPMS) encoded by leuA in Mycobacterium tuberculosis (M.tb) is involved in the leucine biosynthesis pathway and is extremely critical for the synthesis of branched-chain amino acids (leucine, isoleucine and valine). α-IPMS activity is required not only for the proliferation of M.tb but is also indispensable for its survival during the latent phase of infection. It is absent in humans and is widely regarded as one of the validated drug targets against Tuberculosis (TB). Despite its essentiality, any study on designing of potential chemical inhibitors against α-IPMS has not been reported so far. In the present study, in silico identification of putative inhibitors against α-IPMS exploring three chemical databases i.e. NCI, DrugBank and ChEMBL is reported through structurebased drug design and filtering of ligands based on the pharmacophore feature of the actives. In the absence of experimental results of any inhibitor against α-IPMS, a stringent validation of docking results is done by comparing with molecular mechanics/Poisson- Boltzmann surface area (MM/PBSA) calculations by investigating two more proteins for which experimental results are known.

## Background

Tuberculosis (TB) is an infectious disease caused by Mycobacterium tuberculosis (M.tb). Despite the availability of many treatment regimes, the global incidence of TB is still very high and it remains one of the top ten leading causes of death worldwide. 60% of the new cases (out of 10.4 million) reported in 2015 were from India, Indonesia, China, Nigeria, Pakistan and South Africa only, majority of which were due to reactivation of dormant bacilli residing inside the host (latent TB infection). Severity of the TB burden is further aggravated due to increase in the appearance of multidrug-resistant TB (480000 new cases) and rifampicin-resistant TB (100000 new cases) [1]. Above observations underline the urgent need for the development of new effective drugs against the replicative and dormant phase of both resistant and non-resistant strains of M.tb. Targeting the enzymes essential for the survival of bacterium under unfavorable conditions can lead to the successful treatment of the disease.

M.tb synthesizes branched-chain amino acids (leucine, isoleucine and valine) from α-ketoisovalerate (α-KIV) through leucine biosynthesis pathway. This pathway is present in microorganisms and plants but is absent in humans. Transposon mutagenesis experiments and studies using leucine auxotroph have shown leucine biosynthetic pathway is essential for the growth and survival of M.tb [2,3,4]. α-IPMS, encoded by leuA (Rv3710), catalyze the initial step of the leucine biosynthesis pathway, which involves Claisen condensation of acetyl coenzyme-A (acetyl-CoA) and α-ketoisovalerate (α-KIV) into α- isopropylmalate (α-IPM) and coenzyme-A (CoA). This enzyme has been shown to be essential for the growth of the bacterium in various genomics and proteomics experiments [5,6,7]. α-IPMS is also observed to be upregulated in M.tb guinea pig model after 90-days of infection with the bacterium [8]. The essentiality of α- IPMS in M.tb and its absence in humans makes this enzyme an effective target for the development of therapeutics against tuberculosis.

α-IPMS is a homodimeric protein where each monomer unit (644 residues, 70-kDa) consists of N-terminal catalytic domain and Cterminal regulatory domain, separated by linker domains (subdomain-I & subdomain-II) [9]. The N-terminal domain consists of well-conserved TIM barrel ((α/β)8) fold and Cterminal domain consists of an antiparallel six-stranded βαβ sandwich. The active site lies in the TIM barrel domain and leucine binds to the regulatory domain in a non-competitive manner. The enzyme has been shown to be activated by a monovalent ion and a divalent ion is required for the catalytic activity [10]. α-IPMS is regulated by the end product of the pathway i.e. L-leucine through feedback inhibition mechanism.

High-throughput virtual screening (structure-based drug design (SBDD) and ligand-based virtual screening) of prepared chemical libraries against the target protein is a powerful technique for lead identification. Further, refinement of the leads can be done by calculation of binding free energy through molecular dynamics (MD) simulation and molecular mechanics/Poisson- Boltzmann surface area (MM/PBSA) calculations. These techniques have been employed earlier to design potential inhibitors against many targets [11]. In M.tb, two inhibitors of aspartate-semialdehyde dehydrogenase (ASADH; essential enzyme for synthesis of essential amino acids) [12], four inhibitors of maltosyl transferase [13] and two inhibitors of GlgB (α-1, 4-glucan branching enzyme) [14] have been designed in silico using virtual screening, pharmacophore modelling and MD simulation. The efficacy of inhibitors designed against GlgB was further evaluated in vivo and found to hinder the survival of M.tb inside macrophages [14]. These studies support the possibility of identification of inhibitors by applying computational techniques. In the current work, three chemical libraries have been screened against α-IPMS to identify potential inhibitors using a hybrid virtual screening approach i.e. structure-based docking followed by filtering of ligands based on the pharmacophore features of the known active compounds. Further, molecular dynamics simulation has been performed to assess the result of docking and rescoring of ligands based on binding free energy (MM/PBSA) calculation. Eight inhibitors have been identified jointly from DrugBank and ChEMBL that can act as potential inhibitors against α-IPMS. Validation of the results is done by performing docking and MM/PBSA calculations on two different protein targets and comparing the binding free energy results with the experimental binding free energies.

## Methodology

### Virtual Screening

#### Protein Preparation

α-IPMS was crystallized as a asymmetric homo-dimer complexed with α-KIV and Zinc ion (PDB ID: 1SR9) [9]. The dimeric unit has been prepared for molecular docking using Dock Prep Wizard of UCSF-Chimera [15]. α-IPMS requires a divalent metal ion for catalytic activity but zinc is known to cause inhibition of the enzyme, hence while preparing the receptor, zinc ion has been replaced with magnesium [10]. The metal ion is coordinated by three amino acids (Asp81, His285, His287), α-keto and the carboxyl group of α-KIV along with one water molecule (Residue No: 1108 in chain A, PDB ID: 1SR9). Only this water molecule has been retained while preparation of the receptor. All other water molecules have been removed.

#### Chemical Library Selection and Preparation

Three chemical libraries i.e. National Chemical Institute (NCI), ChEMBL and DrugBank have been selected and filtered on the basis of number of heavy atoms present in each compound. Compounds having less than 21 heavy atoms have been retained, as the active site of α-IPMS is small in size [9]. The final set of compounds has been prepared using LigPrep module of SchrÖdinger suite (v2014-4). The ionization state of each of the chemicals has been predicted using Epik (pH range 7±2) and a maximum of 32 stereoisomers has been generated. A positive set of four compounds (α-ketoisovalerate, α-ketovalerate, α- ketobutyrate and pyruvate) [16], which are substrate to the protein, has also been similarly prepared.

#### Molecular Docking to identify potential inhibitors of α-IPMS

The prepared chemical libraries and the positive set have been screened against the active site of α-IPMS to identify the potential inhibitors. Virtual screening has been performed in three phases: initial two phases using DOCK6.7 [17] and final phase using GOLD v5.2 [18]. In the first phase, all the compounds have been screened with less precision and in the second phase, the top 10% compounds from the first phase has been screened with extra precision (max. orientations: 10000, pruning max. orientations: 10000, simplex anchor max. iterations: 1000, simplex grow max. iterations: 1000 are used as the parameters of the runs) using flexible docking of DOCK6.7 program. Grid-based energy function has been used for scoring of ligands. In the third phase of virtual screening, the top 30 compounds (obtained after filtering the chemical library based on pharmacophore properties of known actives which is discussed below) have been re-docked using GOLD5.0 (GA Run=100). Gold Score has been used for scoring of ligands with default input and annealing parameters.

#### Filtering Based on Pharmacophore Features

Filtering of chemicals based on pharmacophore features of the actives has been performed using Phase module of SchrÖdinger suite (v2014-4). Pharmacophore describes the spatial arrangement of features essential for interaction of ligand and receptor. Pharmacophore features have been identified using known actives i.e. α-ketoisovalerate, α-ketovalerate, α-ketobutyrate and pyruvate [16] and then these features have been utilized to filter the ligand library after the second phase of docking.

#### Molecular dynamics simulation and MM/PBSA Rescoring

Each docking pose has been rescored using MD simulation and MM/PBSA calculation. The molecular dynamic simulation of ligand-protein complexes has been performed using AMBER14 software. The ff14SB Amber forcefield was used in the simulation [19]. The protonation states of the proteins have been determined using PROPKA [20]. General AMBER Force Field (GAFF) parameters [21] have been used for the small ligand molecules. All simulations have been performed using explicit TIP3P water box [22] with 10Å padding and system was neutralized using Na+ ions to allow the use of Particle-mesh Ewald (PME) with periodic box. Energy minimization of each system has been performed in 2-phases; first, for the solvent and ion molecules by restraining the protein-ligand complex and second for entire system after removing the restraints. The system has been slowly heated to 300K in six steps. The simulations have been equilibrated for 1ns and 20ns long production run has been performed in constant pressure ensemble for each protein-ligand complex and the data has been collected at every 2ps interval. Only the last 10ns data has been used for MM/PBSA calculation. The binding free energy of the protein-ligand complex has been calculated using the MM/PBSA-1A approach i.e. only simulating the complex forms [23].

## Results and discussion

### Validation of Docking Protocol

Several methods have been reported earlier for validation of docking programs and scoring functions [24]. One of the simplest methods employed in this regard is pose reproducibility whereby a ligand with known conformation and orientation (typically a crystal structure) is re-docked into the protein/target's active site. If the docking program is able to reproduce the pose within a range of preselected root mean square deviation (rmsd) value (rmsd less than 1.5Å or 2Å is preferred), then the docking program is considered to have performed successfully. Scoring follows this step and ranking of poses using a scoring function. The scoring function that accurately ranks the poses based on rmsd value is further selected for the study. Another method commonly utilized is docking of a decoy set of active and inactive compounds, followed by the ranking of ligands. In this method, the docking program is evaluated by its ability to select known actives over inactive decoys.

In this work, the hybrid docking protocol has been validated using a standard dataset of known actives and decoys of Dihydrofolate reductase (DHFR) [25], which is involved in folate biosynthesis. The dataset consists of 410 active conformers and 8367 decoys (total 8777 conformers). The docking runs have been performed using the protocol described in materials and methods against the target protein DHFR. After the first phase of docking using DOCK6.7, the top 10% conformers (877 conformers) from the top-ranked virtual screening hit list have been selected for second phase. The compound library for the second phase consists of 74 actives and 803 decoys. After the second phase of virtual screening and filtering the compound library based on pharmacophore feature of the ligand (Fitness > 1.5), 184 conformers have been retrieved. Further, top 30 compounds have been selected by ranking ligands based on the number of sites matched, fitness and DOCK score. Out of these top 30 conformers, 29 were active conformers which shows that the protocol described above is capable of separating the decoys i.e. negative set from the actives against the target protein DHFR. Hence, this protocol can be further utilized for other targets whose crystal structure is known.

### Molecular Docking of Chemical Libraries and positive sets

The active site of α-IPMS is a small cavity, which is internally lined by hydrophobic residues and externally by polar charged residues. Considering the small cavity size, the filtering of chemical library based on number of heavy atoms resulted in exclusion of large compounds, which may be docked on the surface of the protein and can lead to artificially high scores with their inclusion in top score compounds. Generally, these chemicals are rejected in the post-docking analysis stage. The DOCK score of top ranked compounds from Drugbank database ranged from -73.00 kcal/mol to -67.00 kcal/mol and the predicted binding affinity (X-score) [26] ranged in between -6.70 kcal/mol to -6.20 kcal/mol. The GOLD score of these compounds ranged from 101.00 to 79.00. Similarly, the DOCK score, GOLD score and predicted binding affinity of top ranked compounds from ChEMBL ranged from -98.15 kcal/mol to -77.29 kcal/mol, 107.65 to 87.38 and -7.28 kcal/mol to -6.4 kcal/mol respectively. The DOCK score of positive set (P1-P4) ranged in between -50.98 kcal/mol to -45.15 kcal/mol. The positive set compounds share identical functional group and differ only in the distal portion of the chain. The proximal end is acidic in nature (keto acid) and distal portion is hydrophobic in nature, which differs only in alkyl chain. The MCS tanimoto similarity for the four positive compounds varies from 0.75 to 0.87 and hence we have used pharmacophore features of known actives to filter the compounds rather than similarity. This filtering step using pharmacophore features of the known actives removed 584 compounds from DrugBank, 13978 from NCI and 38546 compounds from ChEMBL respectively. This step ensured the presence of essential features in the filtered compounds. The number of hydrogen bonds for the best docking poses obtained by molecular docking varied from 6 for CHEMBL1615775 to 12 for CHEMBL404748 and the hydrophobic interactions from 22 to 50. Arg80, Tyr169, His285 and His287 are major hydrogen bond forming residues. Except for CHEMBL1162019, Arg80 forms hydrogen bond with the entire top score compounds. The compounds also form hydrogen bonds with the residues of the chain B (Ser376B, Gly377B, Ser378B, His379B, Tyr410B), which are present in the vicinity of the active site. All the 15 compounds have higher GOLD score and lower DOCK score than the positive set and hence these compounds may act as inhibitors against α-IPMS. The compounds of NCI has been discarded because these compounds ranked lower than the positive set and further, these compounds did not form hydrogen bond with the critical residues.

### MM/PBSA rescoring identifies CHEMBL404748 and CHEMBL1159999 as potential inhibitors of α-IPMS.

**Figure 1:**
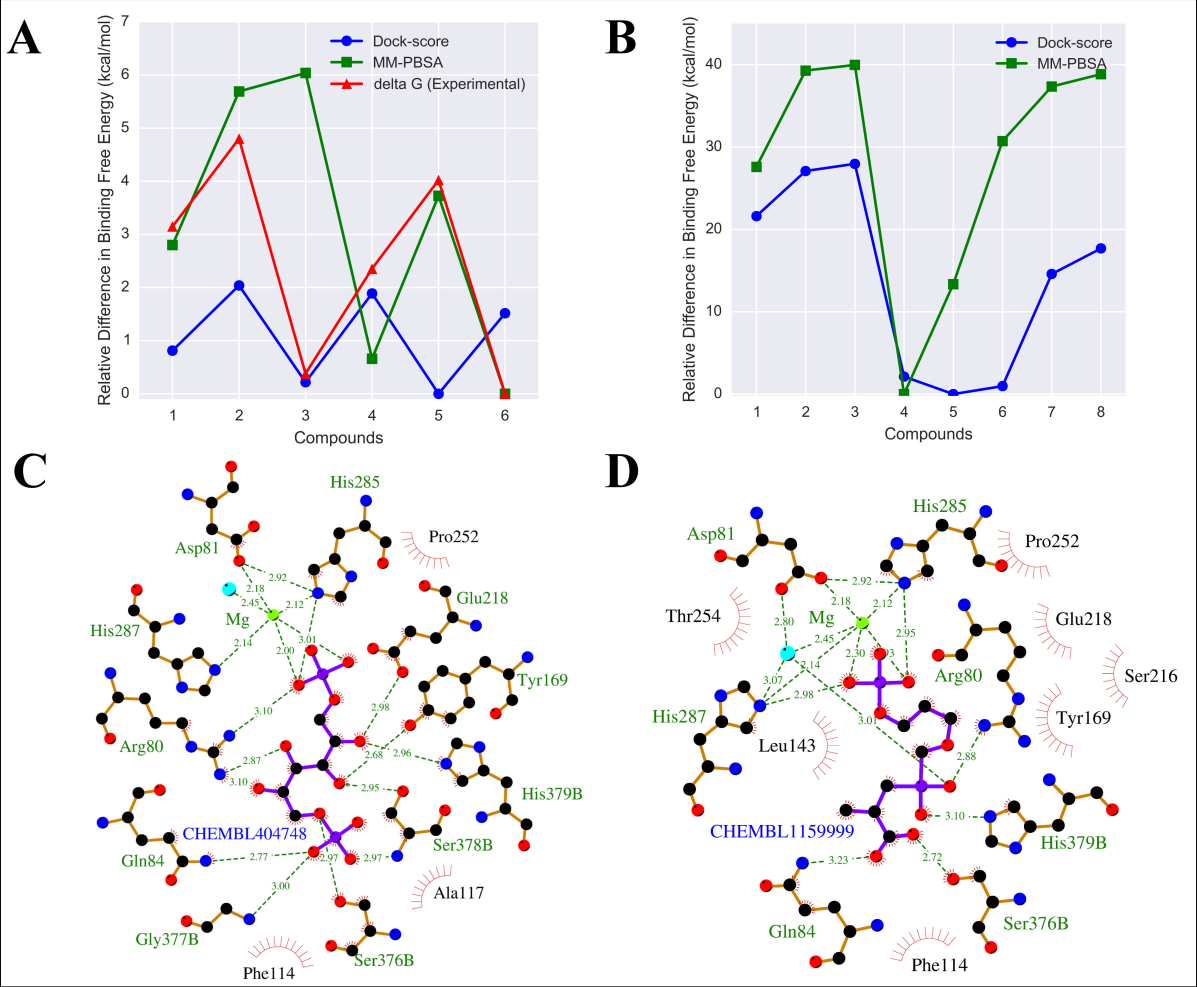
A) Relative difference in dock score and binding free energy for inhibitors of DHFR (1-6 represents DH1-DH6) and comparison with experimental data. B) Relative difference in dock score and binding free energy for proposed inhibitors of α-IPMS (1- 3 represents D1-D3 and 4-8 represents C1-C5 respectively). C) Ligplot of CHEMBL404748. D) Ligplot of CHEMBL1159999. In ligplot, green dashed lines indicate hydrogen bonds and the number indicates the inter-atomic distance in Å. Red arcs with spikes represents hydrophobic interactions. Hydrogen Bond forming residues are labeled in green and residues involved in hydrophobic interactions are labeled in black.

In the final step of our work, MD Simulation has been carried out for all the 15 identified inhibitors through molecular docking and the positive set. Molecular Dynamic Simulation has also been carried out for six known inhibitors of DHFR (DH1-DH6) and six known inhibitors of Catechol-O-methyltransferase (COMT) (CO1-CO6) for benchmarking of MM/PBSA results [27]. The binding free energy of each protein-ligand complex has been calculated using MM/PBSA approach. The results are summarized in Table 1. Figure 1A shows the comparison of binding energy calculated through docking (dock-score) and MM/PBSA with the experimental data for DHFR. It can be seen from the figure that MM/PBSA calculation for inhibitors of DHFR shows fair correlation with the experimental data and correctly identified the inhibitor with the lowest binding free energy. The MM/PBSA calculation for inhibitors of COMT also shows reasonable correlation with the experimental data (Table 1), which shows that MM/PBSA calculations can successfully capture the relative difference in binding free energy.

Now we describe the results of MM/PBSA for a-IPMS. The binding free energy for positive set ranged from -34.43 ± 3.78 kcal/mol (α-ketoisovalerate) to -14.33 ± 3.86 kcal/mol (Pyruvate). The binding free energy of chemicals from DrugBank lie in between -57.37 ± 4.51 kcal/mol to -21.03 ± 5.27 kcal/mol and ChEMBL from -84.97 ± 7.97 kcal/mol to -16.60 ± 8.28 kcal/mol. Several chemicals identified through molecular docking as potential inhibitors failed to rank well in MM/PBSA rescoring as can be seen from Table 1. This can be attributed to lack of elaborate and accurate scoring functions used in docking. Eight compounds, three from DrugBank (DB04182, DB03502, DB04304) and five from ChEMBL (CHEMBL404748, CHEMBL1159999, CHEMBL1235112, CHEMBL1161477 and CHEMBL1615775) ranked better than all the positive sets in MM/PBSA rescoring (Table 1). The relative differences in binding free energy of these eight compounds are shown in Figure 1B. The lowest binding free energy has been obtained for CHEMBL404748 and CHEMBL1159999, which are -84.97 ± 7.97 kcal/mol, and -71.61 ± 4.27 kcal/mol respectively. The binding free energy of these two chemicals is approximately two times lower than the catalytic substrate and other actives, hence these two chemicals can be potential inhibitors; however, further experimental validation is required. The LigPlot [28] of interactions involving ligand i.e. CHEMBL404748 and CHEMBL1159999 with the receptor residues is shown in Figure 1C & 1D. CHEMBL404748 (Glucitol Bis-Phosphate) is a known potential inhibitor of rabbit muscle aldolase (representative of class I aldolases), Helicobacter pylori, and Saccharomyces cerevisiae aldolases (representative of class II Faldolases) [29]. It is to be noted that the solute entropy calculation is not performed in the MM/PBSA calculation, which may change the numerical values of the binding free energy reported. It is expected that some of the proposed compounds will be investigated experimentally to understand their efficacy.

## Conclusion

In this work, an integrated approach has been used to design inhibitors against α-IPMS considering the structural properties of protein and pharmacophore properties of known active ligands. To ensure diverse set of chemical libraries, virtual screening has been performed using three chemical libraries viz. DrugBank, NCI and ChEMBL. The generation of focused library could help in reducing computational time for virtual screening. Altogether, from DrugBank and ChEMBL, eight potential inhibitors of α- IPMS has been found which have relatively better binding affinity than known active compounds, out of which CHEMBL404748 and CHEMBL1159999 are suggested to be the most potent against α-IPMS.

## Figures and Tables

**Table 1:** Molecular docking and MM/PBSA results for inhibitors of DHFR (DH1-DH6), inhibitors of COMT (CO1-CO6) and positive set (P1-P4), inhibitors from DrugBank (D1-D3) & inhibitors from ChEMBL (C1-C5) of α-IPMS.

Id	Drugbank/ ChEMBL/ZINC Id /Name	Dock Score (kcal/mol)	Δ Dock Score ^b^ (kcal/mol)	ΔΔG_exp_^a,b^ (kcal/mol)	ΔΔG_bind_ (MM/PBSA) ^b^ (kcal/mol)
DH1	ZINC03814961	-42.65	0.82	3.15	2.8
DH2	ZINC00006585	-41.43	2.04	4.8	5.69
DH3	ZINC03814951	-43.25	0.22	0.38	6.03
DH4	ZINC03814865	-41.58	1.89	2.35	0.66
DH5	ZINC03814952	-43.47	0	4.02	3.72
DH6	ZINC01489187	-41.95	1.52	0	0
CO1	CHEMBL3425734	-28.54	1.08	0	4.38
CO2	CHEMBL3425743	-26.28	3.34	0.77	9.58
CO3	CHEMBL3425722	-23.26	6.36	1.73	7.98
CO4	CHEMBL3425737	-29.62	0	2.06	0
CO5	CHEMBL3425725	-24.92	4.7	2.55	11.28
CO6	CHEMBL3425728	-26.38	3.24	2.87	11.79
P1	α-ketoisovalerate	-49.74	45.24	---	50.54
P2	α-ketovalerate	-50.98	44	---	58.07
P3	α-ketobutyrate	-49.19	45.79	---	61.75
P4	Pyruvate	-45.15	49.83	---	70.64
D1	DB04182	-73.36	21.62	---	27.6
D2	DB03502	-67.88	27.1	---	39.3
D3	DB04304	-67.01	27.97	---	39.98
C1	CHEMBL404748	-92.85	2.13	---	0
C2	CHEMBL1159999	-94.98	0	---	13.36
C3	CHEMBL1235112	-94.01	0.97	---	30.73
C4	CHEMBL1161477	-80.4	14.58	---	37.36
C5	CHEMBL1615775	-77.29	17.69	---	38.86

a ΔGexp values have been calculated using the formula below : *ΔG=-RTlnk_i_* where, R = 1.9872036 * 10-3 kcal K-1 mol-1, T = 300 K and *k_i_ =IC_50_/(1+S/K_m_) ^b^* Δ values (i.e. Δ Dock Score, ΔΔG_exp_ and ΔΔG_bind_ (MM/PBSA)) have been calculated by subtracting the binding free energy of reference compound (Compound with minimum binding free energy) obtained in each group.
